# Nonstandard Higgs couplings from angular distributions in $h\rightarrow Z\ell ^+\ell ^-$

**DOI:** 10.1140/epjc/s10052-014-2798-2

**Published:** 2014-03-12

**Authors:** Gerhard Buchalla, Oscar Catà, Giancarlo D’Ambrosio

**Affiliations:** 1 Arnold Sommerfeld Center for Theoretical Physics, Fakultät für Physik, Ludwig-Maximilians-Universität München, 80333 Munich, Germany; 2 TUM-IAS, Lichtenbergstr. 2a, 85748 Garching, Germany; 3 Physik Department, TUM, 85748 Garching, Germany; 4 INFN-Sezione di Napoli, Via Cintia, 80126 Naples, Italy

## Abstract

We compute the fully differential rate for the Higgs-boson decay $h\rightarrow Z\ell ^+\ell ^-$, with $Z\rightarrow \ell ^{'+}\ell ^{'-}$. For these processes we assume the most general matrix elements within an effective Lagrangian framework. The electroweak chiral Lagrangian we employ assumes minimal particle content and Standard Model gauge symmetries, but it is otherwise completely general. We discuss how information on new physics in the decay form factors may be obtained that is inaccessible in the dilepton-mass spectrum integrated over angular variables. The form factors are related to the coefficients of the effective Lagrangian, which are used to estimate the potential size of new-physics effects.

## Introduction

The recent discovery of a light scalar $h$ by ATLAS [1] and CMS [2] has been a major step forward in our understanding of electroweak symmetry breaking. The first run of the LHC has established its mass with an accuracy of better than $1\,\%$ and has provided evidence for its scalar nature with spin-parity $0^+$ [3]. Furthermore, decay rates to gauge-boson pairs show no significant deviations from their Standard Model (SM) values [4, 5] within the present accuracy of around 20–30 % [6, 7]. The overall agreement with the Standard Model is so far impressive.

However, theoretical arguments suggest that deviations should be expected. Their absence would actually be rather puzzling and would point to a fine-tuned solution for electroweak symmetry breaking, where the lightness of the Higgs would remain unexplained. Deviations from the Standard Model parameters open the gate to new physics, expected to lie at the Terascale in the form of weakly or strongly coupled new interactions. So far the LHC has been able to test total decay rates of $h$ into gauge-boson pairs. However, LHC run 2, with a substantial increase in luminosity, will provide enough statistics to probe also differential distributions, thereby testing the Standard Model in much greater detail.

In this paper we will study in a model-independent way the impact of new physics in the full angular distribution of $h\rightarrow Z{\ell }^+{\ell }^-$ decay, with the $Z$ on-shell and eventually decaying into a lepton pair. We will argue that $h\rightarrow Z{\ell }^+{\ell }^-$ is a useful channel not only for spin identification [8–12], but also to test nonstandard couplings: it provides a rich 4-body angular distribution with a clean 4-lepton final-state signature. For earlier work see [13, 14].

Our results can be parametrized in terms of six independent dynamical form factors, which include the effects of virtual electroweak bosons ($\gamma$ and $Z$) as well as heavier states, whose effects at the electroweak scale are encoded in contact interactions. Since we aim at model independence, we will study the new physics contributions using the effective field theory (EFT) scheme developed in [15, 16], which is the most general EFT of the electroweak interactions. As opposed to particular models, the resulting set of new-physics coefficients will remain undetermined. However, their natural sizes can still be estimated with the aid of power-counting arguments.

Certain aspects of this decay mode have already been discussed recently [17–19], with a focus on the dilepton-mass distribution. The observation there is that mass distributions can unveil new-physics structures in an otherwise SM-compatible integrated decay rate. This, however, comes at the expense of some fine-tuning in the new-physics parameters. In contrast, by exploiting angular distributions one can identify structures that do not contribute to the integrated decay rate. Thus, one can still be compatible with the SM decay rates without tuning the new-physics parameters.

As opposed to loop-induced processes, such as $h\rightarrow \gamma Z$, $h\rightarrow Z\ell ^+\ell ^-$ does not look a priori like a promising testing ground for new-physics effects. As we will show below, they are expected, at most, at the few $\%$ level in certain observables. $h\rightarrow Z\ell ^+\ell ^-$ is, however, an exceptionally clean decay mode and the natural suppression of new physics can be compensated with statistics. In fact, the LHC running at 14 TeV with an integrated luminosity of 3000 fb$^{-1}$ will potentially be sensitive to new-physics effects in $h\rightarrow Z\ell ^+\ell ^-$. Our analysis also shows that CP-odd effects in $h\rightarrow Z\ell ^+\ell ^-$ are expected only at the per-mille level.

The remainder of this paper will be organized as follows: in Sect. 2 we will derive the full angular distribution for $h\rightarrow Z{\ell }^+{\ell }^-$. Expressions for the dynamical form factors in terms of EFT coefficients will be given in Sect. 3, with a discussion of their expected sizes in both weakly and strongly coupled scenarios. In Sect. 4 we will discuss some selected angular observables. Conclusions are given in Sect. 5, while an appendix with kinematical details is provided for reference.

## Angular distribution for $h\rightarrow Z{\ell }^+{\ell }^-$

We denote the amplitude for the $h\rightarrow Z \ell ^+\ell ^-$ decay of a Higgs boson by $\varepsilon ^\mu {\mathcal M}_{3,\mu }$, and for the decay of an on-shell $Z$-boson into a lepton pair by $\varepsilon ^\mu {\mathcal M}_{2,\mu }$, where $\varepsilon ^\mu$ is the $Z$-boson polarization. The fully differential decay rate for $h(k)\rightarrow Z(p)\ell ^+(q_1)\ell ^-(q_2)$, followed by $Z(p)\rightarrow \ell ^{'+}(p_1)\ell ^{'-}(p_2)$, is then given, in the narrow-width approximation, by

1 $$\begin{aligned} \frac{d\Gamma }{ds\, d\cos \alpha \, d\cos \beta \, d\phi } = \frac{\lambda }{(2\pi )^5\, 2^{10}\, \sqrt{r}\Gamma _Z} \left| {\mathcal M}^\mu _3 {\mathcal M}_{2,\mu }\right| ^2\nonumber \\ \end{aligned}$$

where we have defined

2 $$\begin{aligned} r&= \frac{m^2_Z}{M^2_h}\, ,\quad s=\frac{q^2}{M^2_h} ,\nonumber \\ \lambda&= (1+ r^2 + s^2 - 2r - 2s - 2 r s)^{1/2} \end{aligned}$$

and $\Gamma _Z$ is the total width of the $Z$. The kinematics is further discussed in Appendix A.

For massless leptons the decay amplitudes can be written as ($\epsilon _{0123}=+1$)

3 $$\begin{aligned}&{\mathcal M}_{3,\mu } = i \frac{2^{1/4} G^{1/2}_F r}{s-r} \cdot \bar{u}(q_2)\left[ 2 F_1 \gamma _\mu (G_V - G_A \gamma _5)\right. \nonumber \\&\quad \left. + \frac{q_\mu }{M^2_h} \not \! k (H_V - H_A \gamma _5)+ \frac{\epsilon _{\alpha \mu \beta \lambda }}{M^2_h} p^\alpha q^\beta \gamma ^\lambda (K_V - K_A \gamma _5)\right] \nonumber \\&\quad \times \,\, v(q_1) \end{aligned}$$

and

4 $$\begin{aligned} {\mathcal M}_{2,\mu } = i \bar{u}(p_2)\gamma _\mu (g_V - g_A \gamma _5) v(p_1). \end{aligned}$$

The form of the amplitude in (3) is valid through next-to-leading order (NLO) of the general electroweak effective Lagrangian described in [16] and in Sect. 3. The form factors $G_{V,A}$, $H_{V,A}$, $K_{V,A}$ are functions of $r$ and $s$. The global normalization of the amplitude has been chosen such that in the Standard Model at leading order $F_1\equiv 1$, $G_V=g_V$, and $G_A=g_A$.

Summing over the final-state lepton polarizations gives

5 $$\begin{aligned} \left| {\mathcal M}^\mu _3 {\mathcal M}_{2,\mu }\right| ^2 = \sqrt{2} G_F M^4_h \left( \frac{r}{r-s}\right) ^2 J(r,s,\alpha ,\beta ,\phi ) \end{aligned}$$

where

6 $$\begin{aligned}&J(r,s,\alpha ,\beta ,\phi ) = J_1\frac{9}{40}(1+\cos ^2\alpha \cos ^2\beta )\nonumber \\&\quad +\,\,J_2\frac{9}{16}\sin ^2\alpha \sin ^2\beta + J_3 \cos \alpha \cos \beta \nonumber \\&\quad +\,\,\left( J_4 \sin \alpha \sin \beta \right. \left. + J_5 \sin 2\alpha \sin 2\beta \right) \sin \phi \nonumber \\&\quad +\,\,\left( J_6 \sin \alpha \sin \beta \right. \left. + J_7 \sin 2\alpha \sin 2\beta \right) \cos \phi \nonumber \\&\quad +\,\,J_8 \sin ^2\alpha \sin ^2\beta \sin 2\phi + J_9 \sin ^2\alpha \sin ^2\beta \cos 2\phi . \end{aligned}$$

The previous expression factors out the angular dependence, $J_i$ being dynamical functions which depend only on the invariant masses $r$, $s$. They are given by

7 $$\begin{aligned} J_1&= \frac{640}{9} F^2_1 (G^2_V + G^2_A) (g^2_V + g^2_A) r s\nonumber \\ J_2&= \frac{32}{9} F_1 (g^2_V + g^2_A) \left[ 2 F_1(G^2_V + G^2_A) (\lambda ^2 + 2 r s)\right. \nonumber \\&\left. +\,\,(G_V H_V + G_A H_A)\lambda ^2 (1-r-s)\right] \nonumber \\ J_3&= 128 F^2_1 G_V G_A g_V g_A r s\nonumber \\ J_4&= 8 F_1 (G_V K_A+ G_A K_V)g_V g_A \lambda \sqrt{r s} (1-r-s)\nonumber \\ J_5&= F_1 (G_V K_V + G_A K_A) (g^2_V + g^2_A) \lambda \sqrt{r s} (1-r-s)\nonumber \\ J_6&= -8F_1 g_V g_A \sqrt{r s} \left[ 8 F_1 G_V G_A(1-r-s) \right. \nonumber \\&\left. +(G_V H_A + G_A H_V)\lambda ^2\right] \nonumber \\ J_7&= -F_1 (g^2_V + g^2_A) \sqrt{r s} \left[ 4 F_1(G^2_V + G^2_A) (1-r-s)\right. \nonumber \\&\left. +\,\,(G_V H_V + G_A H_A)\lambda ^2\right] \nonumber \\ J_8&= -4 F_1 (G_V K_V + G_A K_A) (g^2_V+ g^2_A) \lambda r s\nonumber \\ J_9&= 8 F^2_1 (G^2_V + G^2_A) (g^2_V + g^2_A) r s. \end{aligned}$$

As will be explained in more detail in Sect. 3, the form factors $G_{V,A}$ receive leading-order contributions in the Standard Model, whereas $H_{V,A}$ and $K_{V,A}$ only arise as next-to-leading-order corrections and capture, respectively, CP-even and CP-odd contributions. In writing the expression for the $J_i$, we have therefore consistently neglected terms of second order in $H_{V,A}$ and $K_{V,A}$. It follows that to leading order in the Standard Model the observables $J_4$, $J_5$, and $J_8$, which carry the dependence on $K_{V,A}$, are zero, as one would expect from general CP considerations.

With sufficient data, a general fit to the angular distribution of the four final-state leptons could in principle extract all nine terms $J_i$ in the fully differential decay rate (1), (5) and (6). From (7) we see that measuring $J_1$, $\ldots$, $J_6$, for example, would determine the 6 independent combinations

8 $$\begin{aligned} \begin{array}{l@{\quad }l} G^2_V+ G^2_A, &{}G_V G_A\\ G_V H_V + G_A H_A,&{}G_V H_A + G_A H_V\\ G_V K_V + G_A K_A,&{}G_V K_A + G_A K_V \end{array}. \end{aligned}$$

All of the six form factors $G_{V,A}$, $H_{V,A}$, $K_{V,A}$ could then be obtained. The remaining three observables $J_7$, $J_8$, and $J_9$ give no independent information on these form factors. They can be used for a cross-check or as alternative input. The coefficients $g_{V,A}$ are not directly related to the process $h\rightarrow Z\ell ^+\ell ^-$ and have to be constrained independently from properties of $Z$ decays.

With limited data, it is more efficient to extract the different $J_i$ projecting them from (6). Integrating the distribution in (6) over $\phi$ we are left with $J_1$, $J_2$, and $J_3$ as the only observables. Integrating in addition over $\alpha$ and $\beta$ eliminates $J_3$. Thus, the differential rate $d\Gamma /ds$, fully integrated over the angular variables, remains sensitive only to $J_1+J_2$. Performing the angular integrations one obtains the dilepton-mass spectrum of the $h\rightarrow Z\ell ^+\ell ^-$ rate, multiplied by the $Z\rightarrow \ell ^{'+}\ell ^{'-}$ branching fraction $B_\ell$. From (1) one finds

9 $$\begin{aligned} \frac{d\Gamma }{ds}&= B_\ell \, \frac{G_F M^3_h}{\sqrt{2}\, 192\pi ^3} \frac{\lambda r}{(r-s)^2}\nonumber \\&\times \,\,F_1 \left[ 2 F_1(G^2_V + G^2_A) (\lambda ^2 + 12 r s)\right. \nonumber \\&\left. +\,\,(G_V H_V + G_A H_A)\lambda ^2 (1-r-s)\right] \end{aligned}$$

where

10 $$\begin{aligned} B_\ell =\frac{(g^2_V + g^2_A) m_Z}{12\pi \Gamma _Z}. \end{aligned}$$

In contrast, $J_3$, $\ldots$, $J_9$ have to be accessed with appropriate angular asymmetries. For instance, the term $J_3$ can be extracted by integrating over $\phi$ and forming a suitable forward–backward asymmetry in $\cos \alpha$ and $\cos \beta$. In Sect. 4 we examine this and other angular asymmetries in detail.

The angular distribution in $h\rightarrow Z\ell ^+\ell ^-$ is similar to the one in the rare $B$-meson decay $B\rightarrow K^*\ell ^+\ell ^-$, which has been discussed for instance in [20–23]. However, in the present case the angles $\alpha$ and $\beta$ are on an equal footing, and accordingly the angular dependence in (6) is symmetric under the interchange of $\alpha$ and $\beta$. Note in particular that the forward–backward asymmetry term $J_3$ is proportional to the product $\cos \alpha \, \cos \beta$, thus representing a kind of correlated double asymmetry in $\alpha$ and $\beta$. It vanishes when either $\alpha$ or $\beta$ are integrated over their full range. This is in contrast to $B\rightarrow K^*\ell ^+\ell ^-$, where a forward–backward asymmetry in the single angle $\alpha$ exists due to the more complicated structure of the hadronic transition $B\rightarrow K^*$.

## Form factors from effective Lagrangian

In order to estimate the form factors $G_{V,A}$, $H_{V,A}$, $K_{V,A}$ in (3) and $g_{V,A}$ in (4) we will work with the nonlinear effective Lagrangian discussed in [15, 16]. A subset of the relevant operators has also been discussed in [24, 25]. In this framework, electroweak symmetry breaking is realized by spontaneously breaking a global $SU(2)_L\times SU(2)_R$ down to $SU(2)_V$. The resulting Goldstone modes are then collected into a matrix $U$ transforming as $g_L U g_R^{\dagger }$ under the global group. One also defines

11 $$\begin{aligned} D_\mu U=\partial _\mu U+i g W_\mu U -i g' B_\mu U T_3 \end{aligned}$$

such that the SM subgroup $SU(2)_L\times U(1)_Y$ is gauged. For convenience we will use the shorthand notation

12 $$\begin{aligned} L_{\mu }=iUD_{\mu }U^{\dagger },\qquad \tau _L=UT_3U^{\dagger } \end{aligned}$$

for the Goldstone covariant derivative and the custodial symmetry breaking spurion $T_3$. The Higgs field $h$ is introduced as an additional light (pseudo-Goldstone) boson, singlet under the SM gauge group.

With these definitions one has at leading order [16, 26, 27]

13 $$\begin{aligned} {\mathcal {L}}_{LO}&= -\frac{1}{2}\langle W_{\mu \nu }W^{\mu \nu }\rangle -\frac{1}{4} B_{\mu \nu }B^{\mu \nu }+ i\!\sum _{f}\bar{\psi }_f \!\not \!\! D\psi _f\nonumber \\&+\frac{v^2}{4}\ \langle L_\mu L^\mu \rangle f\left( \frac{h}{v}\right) -\frac{1}{2}h(\partial ^2+M^2_h)h - V(h).\nonumber \\ \end{aligned}$$

For $h\rightarrow Z{\ell }^+{\ell }^-$ the final-state fermions can be taken massless to an excellent approximation and therefore we have omitted the Yukawa terms above. The main contribution to $h\rightarrow Z\ell ^+\ell ^-$ comes from the subprocess $h\rightarrow ZZ^*$, which is described by the gauge-boson mass term, where $f(h/v)$ can be truncated at linear order for the process of interest here:

14 $$\begin{aligned} f\left( \frac{h}{v}\right) =1+2a\frac{h}{v}. \end{aligned}$$

At next-to-leading order (NLO) there are eight relevant CP-even operators

15 $$\begin{aligned}&{\mathcal {O}}_{Xh1}=g^{\prime 2} B_{\mu \nu }B^{\mu \nu }\frac{h}{v} f_{Xh1}\left( \frac{h}{v}\right) ,\nonumber \\&{\mathcal {O}}_{Xh2}=g^2\langle W_{\mu \nu }W^{\mu \nu }\rangle \frac{h}{v} f_{Xh2}\left( \frac{h}{v}\right) ,\nonumber \\&{\mathcal {O}}_{XU1}=g^{\prime }gB_{\mu \nu }\langle W^{\mu \nu }\tau _L\rangle f_{XU1}\left( \frac{h}{v}\right) ,\nonumber \\&{\mathcal {O}}_{XU2}=g^2 \langle W^{\mu \nu }\tau _L\rangle ^2 f_{XU2}\left( \frac{h}{v}\right) ,\nonumber \\&{\mathcal {O}}_{V7}=-{\bar{l}}\gamma _{\mu }l\langle \tau _LL^{\mu }\rangle f_{V7}\left( \frac{h}{v}\right) ,\nonumber \\&{\mathcal {O}}_{V8}=-{\bar{l}}\gamma _{\mu }\tau _L l\langle \tau _LL^{\mu }\rangle f_{V8}\left( \frac{h}{v}\right) ,\nonumber \\&{\mathcal {O}}_{V10}=-{\bar{e}}\gamma _{\mu }e\langle \tau _LL^{\mu }\rangle f_{V10}\left( \frac{h}{v}\right) ,\nonumber \\&{\mathcal {O}}_{\beta _1}= -v^2\langle \tau _LL_{\mu }\rangle ^2f_{\beta _1}\left( \frac{h}{v}\right) , \end{aligned}$$

and four CP-odd ones:

16 $$\begin{aligned} {\mathcal {O}}_{Xh4}&= g^{\prime 2} \epsilon _{\mu \nu \lambda \rho }B^{\mu \nu } B^{\lambda \rho } \frac{h}{v} f_{Xh3}\left( \frac{h}{v}\right) ,\nonumber \\ {\mathcal {O}}_{Xh5}&= g^2\epsilon _{\mu \nu \lambda \rho }\langle W^{\mu \nu } W^{\lambda \rho }\rangle \frac{h}{v} f_{Xh4}\left( \frac{h}{v}\right) ,\nonumber \\ {\mathcal {O}}_{XU4}&= g^{\prime }g \epsilon _{\mu \nu \lambda \rho } B^{\mu \nu } \langle W^{\lambda \rho }\tau _L\rangle f_{XU4}\left( \frac{h}{v}\right) ,\nonumber \\ {\mathcal {O}}_{XU5}&= g^2\epsilon _{\mu \nu \lambda \rho } \langle \tau _L W^{\mu \nu }\rangle \langle \tau _L W^{\lambda \rho }\rangle f_{XU5}\left( \frac{h}{v}\right) . \end{aligned}$$

Some comments are in order:

- Fermionic tensor operators are in principle also present, but they turn out to be negligible: first, they have a chiral suppression and second, they do not interfere with the Standard Model and thus can only appear at NNLO.
- For simplicity, the list above includes only fermions of the first family. The extension to include the second family is, however, trivial.
- The $f_i(h/v)$ above are generic functions with model-dependent coefficients [16]. As a result, the previous operators contain all the possible powers of $h$. In the following, $a_i$ and $b_i$ will denote, respectively, the dimensionless Wilson coefficients for the pieces without $h/v$ and linear in $h/v$, which are the relevant ones for the process under study.

**Fig. 1:**
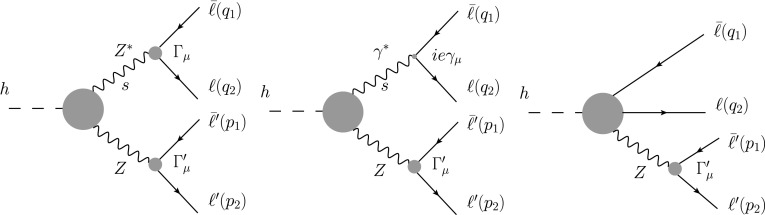
Different contributions to $h\rightarrow Z\ell ^+\ell ^-$

The operators above give the most general direct contributions to the vertices of Fig. 1, but they also lead to a renormalization of the fields and parameters [28, 29]. These effects will be consistently included in all our results. As the fundamental electroweak parameters we will employ $\alpha _{em}=e^2/4\pi$, $m_Z$ and $G_F$ ($Z$-standard definition). Then the NLO corrections can finally be expressed in terms of the following effective interactions:

17 $$\begin{aligned} {\mathcal {L}}_{NLO}&= 2^{1/4} G^{1/2}_F m_Z^2F_1\, h\, Z^{\mu }Z_{\mu }+b_2 \frac{h}{v}Z^{\mu \nu }Z_{\mu \nu }\nonumber \\&+b_2^{\gamma }\frac{h}{v}Z^{\mu \nu }A_{\mu \nu }+b_3 \frac{h}{v} \epsilon _{\mu \nu \lambda \rho } Z^{\mu \nu } Z^{\lambda \rho }\nonumber \\&+b_3^{\gamma }\frac{h}{v} \epsilon _{\mu \nu \lambda \rho } Z^{\mu \nu }A^{\lambda \rho }+Z_\mu {\bar{l}}\gamma ^{\mu }\left[ g_V - g_A\gamma _5\right] l\nonumber \\&+ \frac{h}{v} Z_\mu {\bar{l}}\gamma ^{\mu }\left[ h_V - h_A\gamma _5\right] l. \end{aligned}$$

For convenience, we have defined

18 $$\begin{aligned} g_V&= \frac{g}{4 c_Z}(\kappa _1 - 4 s^2_Z\kappa _2),\nonumber \\ g_A&= \frac{g}{4 c_Z}\kappa _1 \end{aligned}$$

such that at leading order in the Standard Model $\kappa _i=1$. Here $s_Z$ ($c_Z$) denotes the sine (cosine) of the Weinberg angle in the $Z$-standard definition ($\alpha =\alpha (m_Z)$)

19 $$\begin{aligned} s^2_Z c^2_Z\equiv \frac{\pi \alpha }{\sqrt{2} G_F m^2_Z} \end{aligned}$$

and $g$ is the $SU(2)_L$ gauge coupling, where $g s_Z = e=\sqrt{4\pi \alpha }$. By analogy to (18) we have defined

20 $$\begin{aligned} h_V&= \frac{g}{4 c_Z}(\omega _1 - 4 s^2_Z\omega _2),\nonumber \\ h_A&= \frac{g}{4 c_Z}\omega _1. \end{aligned}$$

In terms of the coefficients of (17), the form factors read

21 $$\begin{aligned} G_V&= g_V \left( 1-\frac{b_2}{F_1}\frac{1-r-s}{r}\right) -\frac{b_2^{\gamma } e}{2 F_1}\frac{(1-r-s)(s-r)}{r s}\nonumber \\&+ \frac{h_V}{2 F_1} \frac{s-r}{r},\nonumber \\ G_A&= g_A \left( 1-\frac{b_2}{F_1}\frac{1-r-s}{r}\right) + \frac{h_A}{2 F_1} \frac{s-r}{r},\nonumber \\ H_V&= \frac{4 b_2}{r} g_V + 2 b_2^{\gamma } e\frac{s-r}{r s},\nonumber \\ H_A&= \frac{4 b_2}{r} g_A,\nonumber \\ K_V&= -\frac{8 b_3}{r} g_V - 4 b_3^{\gamma } e\frac{s-r}{r s},\nonumber \\ K_A&= -\frac{8 b_3}{r} g_A. \end{aligned}$$

In turn, the operators in the Lagrangian (17) can be expressed in terms of the basic EFT operators listed before. For the coefficients this implies ($t_Z=s_Z/c_Z$),

22 $$\begin{aligned}&F_1=a(1+ 2\beta _1 -\delta _G) - b_{\beta _1},\nonumber \\&b_{2,3}\!=\!\frac{e^2}{2}\left( 2t_Z^2b_{Xh1,4}\!+\!t_Z^{-2}b_{Xh2,5}-b_{XU1,4}\!+\! \frac{t_Z^{-2}}{2}b_{XU2,5}\right) \!,\nonumber \\&b_{2,3}^{\gamma }=e^2\left( -2t_Z b_{Xh1,4}+t_Z^{-1}b_{Xh2,5}\right. \nonumber \\&\qquad \quad \,\,\left. +\frac{1}{2}(t_Z^{-1} -t_Z) b_{XU1,4}\right. \left. +\frac{t_Z^{-1}}{2}b_{XU2,5}\right) , \end{aligned}$$

and

23 $$\begin{aligned} \kappa _1&\equiv 1-a_{V7}+\frac{1}{2} a_{V8}+ a_{V10}+ \beta _1-\delta _G ,\nonumber \\ \kappa _2&\equiv 1+\frac{1}{2s^2_Z} a_{V10}+\frac{\delta _G-\beta _1 - a_{XU1} e^2/s^2_Z}{c^2_Z - s^2_Z},\nonumber \\ \omega _1&\equiv -b_{V7}+\frac{1}{2} b_{V8} + b_{V10},\nonumber \\ \omega _2&\equiv \frac{1}{2s^2_Z} b_{V10}. \end{aligned}$$

For simplicity, in (23) we have dropped the family indices, but one should keep in mind that the NLO corrections are in general different for electron and muon final states. Incidentally, notice that $\kappa _i$ also contain a universal (family-independent) contribution, proportional to $\beta _1$, $\delta _G$, and $a_{XU1}$, which results from taking into account the NLO renormalization effects. $\delta _G$ above stands for the renormalization of the Fermi constant, which includes 4-fermion operators not listed in (15). More details can be found in [29].

For completeness we will also discuss the weakly coupled case using the EFT developed in [30] using the notation of [31] (for different approaches see [32, 33]). The relevant operators are now

24 $$\begin{aligned}&{\mathcal {O}}_{HB}= g^{\prime 2}B_{\mu \nu }B^{\mu \nu } H^{\dagger }H,\nonumber \\&{\mathcal {O}}_{HW}=g^2\langle W_{\mu \nu }W^{\mu \nu }\rangle H^{\dagger }H,\nonumber \\&{\mathcal {O}}_{HWB}=gg^{\prime }H^{\dagger }W_{\mu \nu } H B^{\mu \nu },\nonumber \\&{\mathcal {O}}_{HD}=|H^{\dagger }D_{\mu }H|^2,\nonumber \\&{\mathcal {O}}_{Hl}^{(1)}=(H^{\dagger }i\mathop {D}\limits ^{\leftrightarrow }_{\mu }H) ({\bar{l}} \gamma ^{\mu }l),\nonumber \\&{\mathcal {O}}_{Hl}^{(3)}=(H^{\dagger }i\mathop {D^a}\limits ^{\leftrightarrow }_{\!\!\!\!\mu }H) ({\bar{l}}\gamma ^{\mu }\tau _a l),\nonumber \\&{\mathcal {O}}_{He}=(H^{\dagger }i\mathop {D}\limits ^{\leftrightarrow }_{\mu }H) ({\bar{e}}\gamma ^{\mu }e),\nonumber \\&{\mathcal {O}}_{H\Box }=(H^{\dagger }H)\Box (H^{\dagger }H), \end{aligned}$$

and ($${\tilde{X}}_{\mu \nu }=\epsilon _{\mu \nu \lambda \rho }X^{\lambda \rho }$$)

25 $$\begin{aligned}&{\mathcal {O}}_{H{\tilde{W}}}=g^2\langle {\tilde{W}}_{\mu \nu }W^{\mu \nu }\rangle H^{\dagger }H,\nonumber \\&{\mathcal {O}}_{H{\tilde{B}}}=g^{\prime 2}{\tilde{B}}_{\mu \nu }B^{\mu \nu } H^{\dagger }H,\nonumber \\&{\mathcal {O}}_{H{\tilde{W}}B}=gg^{\prime }H^{\dagger }{\tilde{W}}_{\mu \nu }H B^{\mu \nu } \end{aligned}$$

for the CP-even and CP-odd sectors, respectively. The effect of ${\mathcal {O}}_{H\Box }$ is to renormalize the Higgs kinetic term. This shift can be absorbed by a field redefinition of $H$, which then affects the $H\rightarrow ZZ$ coupling. This is of no relevance for the distributions but affects the global normalization of the decay [34]. For comparison with the nonlinear case it is convenient to define ${\bar{\alpha }}_j=v^2\alpha _j$. The result reads

26 $$\begin{aligned}&F_1=\left( 1+{\bar{\alpha }}_{H\Box }\right. \left. +\frac{{\bar{\alpha }}_{HD}}{4}-\delta _G\right) ,\nonumber \\&b_{2,3}=\frac{e^2}{2}\left( 2t_Z^2{\bar{\alpha }}_{HB,H{\tilde{B}}}\right. \left. +t_Z^{-2}{\bar{\alpha }}_{HW,H{\tilde{W}}}+{\bar{\alpha }}_{HWB,H{\tilde{W}}B}\right) ,\nonumber \\&b_{2,3}^{\gamma }=e^2\left( -2t_Z {\bar{\alpha }}_{HB,H{\tilde{B}}}\right. +t_Z^{-1}{\bar{\alpha }}_{HW,H{\tilde{W}}}\nonumber \\&\qquad \quad \,\,\left. -\frac{1}{2}(t_Z^{-1}-t_Z){\bar{\alpha }}_{HWB,H{\tilde{W}}B}\right) \end{aligned}$$

and

27 $$\begin{aligned} \kappa _1&\equiv 1+({\bar{\alpha }}_{Hl1}+{\bar{\alpha }}_{Hl3}-{\bar{\alpha }}_{He})- \frac{{\bar{\alpha }}_{HD}}{4}-\delta _G,\nonumber \\ \kappa _2&\equiv 1-\frac{1}{2s^2_Z} {\bar{\alpha }}_{He}+\frac{1}{c^2_Z-s^2_Z}\left( \frac{{\bar{\alpha }}_{HD}}{4}\right. \left. +e^2\frac{{\bar{\alpha }}_{HWB}}{2s_Z^2}+\delta _G\right) \nonumber \\ \omega _1&\equiv 2({\bar{\alpha }}_{Hl1}+ {\bar{\alpha }}_{Hl3}-{\bar{\alpha }}_{He}),\nonumber \\ \omega _2&\equiv -\frac{1}{s^2_Z} {\bar{\alpha }}_{He}. \end{aligned}$$

It is worth noting that, while the contributions to $h Z\ell ^+\ell ^-$ and $Z \ell ^+\ell ^-$, encoded in $\omega _i$ and $\kappa _i$, respectively, come from the same (family-dependent) NLO operators, $\kappa _i$ also receives a universal NLO renormalization through ${\mathcal {O}}_{HD}$, ${\mathcal {O}}_{HWB}$, and the operators associated with $\delta _G$. Therefore, the contact term contribution to $h\rightarrow Z\ell ^+\ell ^-$ is in general uncorrelated to $Z\rightarrow \ell ^+\ell ^-$, even in the case of the linearly realized Higgs sector. Similarly, the $Z$ mass term and the $h\rightarrow ZZ$ vertex come from the same LO operator but NLO corrections renormalize them differently. As a result, $\delta F_1\ne 0$ in (26).

## Observables and form factor determination

In Sect. 2 we pointed out that at NLO there are six independent form factors entering the dynamical functions $J_i$. With high enough statistics one can fit the full distribution $J$ to experimental data. However, at least in the first stages of the run 2 at the LHC, where statistics will be rather limited, it is more efficient to devise a set of observables that can project out the different form factor combinations through angular asymmetries.

A possible strategy is to extract $G_VG_A$ from the forward–backward asymmetry $A_{\alpha \beta }$ in $\alpha$ and $\beta$, after integration over $\phi$:

28 $$\begin{aligned} A_{\alpha \beta }&= \left( \frac{d\Gamma }{ds}\right) ^{-1} \int \limits _{-1}^{1} d\!\cos \alpha \, {\mathrm {sgn}}(\cos \alpha )\nonumber \\&\int \limits _{-1}^{1}d\!\cos \beta \, {\mathrm {sgn}}(\cos \beta )\displaystyle \frac{d\Gamma }{ds\, d\!\cos \alpha \, d\!\cos \beta }\nonumber \\&= \frac{J_3}{J_1+J_2} \end{aligned}$$

and $(G_V^2+G_A^2)$ from an asymmetry $A_\phi$ in the angle $\phi$:

29 $$\begin{aligned} A_{\phi }&= \left( \frac{d\Gamma }{ds}\right) ^{-1}\int \limits _0^{2\pi } d\phi \, {\mathrm {sgn}}(\cos 2\phi ) \displaystyle \frac{d\Gamma }{dsd\phi }=\frac{32}{9\pi }\frac{J_9}{J_1+J_2}.\nonumber \\ \end{aligned}$$

Knowing $A_{\alpha \beta }$ and $A_{\phi }$, $H_{V,A}$ can be determined through the combinations $g_VH_V+g_AH_A\simeq g_AH_A$ and $g_VH_A+g_AH_V\simeq g_AH_V$. These can be extracted, respectively, from the total rate given in (9) and the asymmetry $B_\phi$,

30 $$\begin{aligned} B_{\phi }=\left( \frac{d\Gamma }{ds}\right) ^{-1}\int \limits _0^{2\pi } d\phi \, {\mathrm {sgn}}(\cos \phi ) \displaystyle \frac{d\Gamma }{dsd\phi }=\frac{\pi }{2}\frac{J_6}{J_1+J_2}.\nonumber \\ \end{aligned}$$

The observables discussed so far test new physics in the CP-even sector. CP-odd contributions are parametrized by $K_{V,A}$, which can be determined through the structures $g_VK_V+g_AK_A\simeq g_AK_A$ and $g_VK_A+g_AK_V\simeq g_AK_V$. They can be extracted from 2 additional asymmetries in $\phi$:

31 $$\begin{aligned} C_{\phi }&= \left( \frac{d\Gamma }{ds}\right) ^{-1}\int \limits _0^{2\pi } d\phi \, {\mathrm {sgn}}(\sin 2\phi )\displaystyle \frac{d\Gamma }{dsd\phi }=\frac{32}{9\pi }\frac{J_8}{J_1+J_2}, \nonumber \\ D_{\phi }&= \left( \frac{d\Gamma }{ds}\right) ^{-1}\int \limits _0^{2\pi } d\phi \, {\mathrm {sgn}}(\sin \phi )\displaystyle \frac{d\Gamma }{dsd\phi }=\frac{\pi }{2}\frac{J_4}{J_1+J_2}.\nonumber \\ \end{aligned}$$

Similar CP-odd observables have been discussed previously in the literature [35–38].

In order to assess the experimental relevance of these asymmetries, we will rely on numerical estimates of new-physics effects based on general power-counting arguments. Accordingly, one would naively expect the NLO coefficients given in the previous section to be generically of ${\mathcal {O}}(v^2/\Lambda ^2)$, with $\Lambda \sim 4\pi v$. Therefore, keeping track of the gauge couplings, we will assume $F_1=a+{\mathcal {O}}(v^2/\Lambda ^2)$, $g_{V,A}=g_{V,A}^{(0)}+g{\mathcal {O}}(v^2/\Lambda ^2)$, $b_{2,3}^{(\gamma )}\sim e^2{\mathcal {O}}(v^2/\Lambda ^2)$, and $h_{V,A}\sim g {\mathcal {O}}(v^2/\Lambda ^2)$.

The main source of deviations from the SM comes from $a$ in $F_1$. This parameter measures the signal strength of $h\rightarrow ZZ^*$, and it is currently constrained to deviate less than $20\,\%$ from the SM. Since our conclusions will be independent of it, we will set $a=1$ and $F_1=1$ for simplicity. New-physics corrections are then naturally dominated by $\delta g_{V,A}$ and $h_{V,A}$. $\delta g_{V,A}$ are constrained by the $Z$ partial width and LEP data sets bounds on them at the $10^{-3}$ level [33, 39], which is within the EFT expectation. $h_{V,A}$ are instead unconstrained, and might in principle attain values larger than the naive EFT dimensional estimate because of numerical enhancements. Consider, for instance, the local $h\rightarrow Z\ell ^+\ell ^-$ couplings $h_{V,A}$ to be induced by the tree-level exchange of a composite heavy vector resonance $R$, mediating $h\rightarrow Z R^*$, $R^*\rightarrow \ell ^+\ell ^-$. Then $h_{V,A}\sim v^2/M^2_R\sim v^2/\Lambda ^2$. If $M_R$ is numerically smaller than $\Lambda \approx 3\,\mathrm{TeV}$ by a factor of three, say, the resulting value of $h_{V,A}$ might be 5–10 times bigger than the naive EFT estimate. This assumes consistency with other phenomenological constraints, which is plausible in view of the free parameters in this scenario.

For simplicity we will consider a scenario where $h_{V,A}\ne 0$, with all other corrections set to zero. Due to the smallness of $g_V$ in the SM, the most sensitive probes of new physics are those linear in $G_V$, namely $A_{\alpha \beta }$ and $B_{\phi }$, with corrections that can easily reach 50–100 %. Incidentally, notice that neither $A_{\alpha \beta }$ nor $B_{\phi }$ are constrained by the angular distributions collected for the spin-parity analysis [3]. This has to be compared with the mass distribution, with typical corrections of a few $\%$. However, both corrections are uncorrelated. Qualitatively, $h_V$ controls $A_{\alpha \beta }$ and $B_{\phi }$ while $h_A$ affects the mass distribution. Thus, one can get large corrections on the former while barely affecting the latter.

**Fig. 2:**
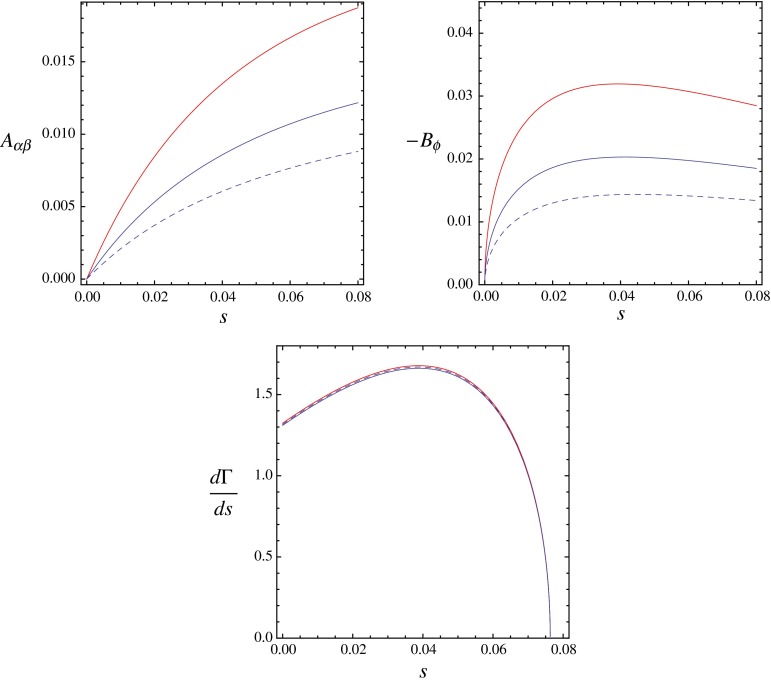
Values for the angular asymmetries $A_{\alpha \beta }$ and $B_{\phi }$ defined in the main text. The *dashed line* corresponds to the SM prediction, while the *solid lines* incorporate potential new-physics effects for the parameter choices $(h_V,h_A)=v^2/\Lambda ^2(-2,0.3)$ (in *blue*) and $(h_V,h_A)=v^2/\Lambda ^2(-6,0.3)$ (in *red*). For comparison, the *lower panel* shows the differential mass distribution (in units of $10^{-6}$ GeV). The plots illustrate the high sensitivity of the angular asymmetries to new physics for scenarios where the mass distribution is left almost unaffected

The reason for this is the accidental suppression of $g_V$ relative to $g_A$ in the standard model by about an order of magnitude. A typical correction from $h_V$ therefore has a larger impact on $G_V$ than $h_A$ has on $G_A$; see eq. (21). Asymmetries that are proportional to $G_{V} G_{A}$ are then rather sensitive to $h_V$. On the other hand, the mass distribution is governed by $G^2_V + G^2_A$, which is approximately given by $G^2_A$ and thus mainly affected by $h_A$. Whereas $h_V$ and $h_A$ are parametrically of the same order of magnitude, we find it interesting for illustration to consider a situation where $h_V$ is numerically enhanced over $h_A$. This could be realized in scenarios with a vector resonance that is lighter than the lowest axial vector state. In Fig. 2 we illustrate such scenarios for the parameter choices $(h_V,h_A)=v^2/\Lambda ^2(-2,0.3)$ and $(h_V,h_A)=v^2/\Lambda ^2(-6,0.3)$.

With the LHC running at 14 TeV and with an integrated luminosity of 3000 fb$^{-1}$, one expects around 6400 reconstructed events for $h\rightarrow Z\ell ^+\ell ^-$ [40]. With such statistics one could in principle reach a 1–2 % sensitivity in the observables that we are discussing. Since the overall effects for $A_{\alpha \beta }$ and $B_{\phi }$ lie around the $\%$ level, as illustrated in Fig. 2, they could be accessible at the LHC, at least in its final stage. Regarding the CP-odd sector, within the range of validity of our EFT, the asymmetries $C_{\phi }$ and $D_{\phi }$ are expected to be below the per-mille level and thus clearly out of reach for detection at the LHC.

These estimates could be made more precise by analysing the size of the backgrounds associated to the specific angular dependences. Such an analysis goes beyond the scope of the present paper, but naively they should be substantially reduced as compared to the total decay rate [11, 41–43]. In this case, $A_{\phi }$ might turn out to be especially suited to extract $(G_V^2+G_A^2)$ with higher precision than through the total decay rate.

Before closing this section, one should note that, strictly speaking, the form factors $G_i,H_i,K_i$ always appear in combination with $g_{V,A}$ in the products

32 $$\begin{aligned} \begin{array}{l@{\quad }l} (G_V^2+G_A^2)(g_V^2+g_A^2),&{}(G_VG_A)(g_Vg_A),\\ (G_VH_V+G_AH_A)(g_V^2+g_A^2),&{}(G_VH_A+G_AH_V)(g_Vg_A),\\ (G_VK_V+G_AK_A)(g_V^2+g_A^2),&{} (G_VK_A+G_AK_V)(g_Vg_A),\\ \end{array}\nonumber \\ \end{aligned}$$

which account for the processes $h\rightarrow Z\ell ^+\ell ^-$ and $Z\rightarrow \ell ^{\prime +}\ell ^{\prime -}$, respectively. In order to determine $G_i,H_i,K_i$ with a certain precision, $g_{V,A}$ should be known comparably well. Unfortunately, with LEP data the bounds on $g_V$ and $g_A$ are too loose to be informative [39]. In contrast, the ILC could offer a clean determination of the $Ze^+e^-$ couplings, since the center-of-mass enhanced corrections to $W^+W^-$ production can be cast entirely in terms of these corrections [29]. As a result, they get singled out at high energies and, within the ILC energy-range, they can naturally be boosted to a $20\,\%$ correction to the production cross section. An analogous mechanism for $Z\mu ^+\mu ^-$ couplings could in principle be pursued in a muon linear collider through $\mu ^+\mu ^-\rightarrow W^+W^-$.

## Conclusions

We have studied, in a general and systematic way, how the decay $h\rightarrow Z\ell ^+\ell ^-$ can be used to probe for physics beyond the Standard Model in the Higgs sector. For this purpose we have employed a general parametrization of the amplitude in terms of form factors, neglecting lepton masses. In view of the large gap between the electroweak scale and the expected scale of new physics, an effective field theory approach appears to be the most efficient tool. We have computed the form factors in terms of the coefficients of an effective Lagrangian, which is defined by the SM gauge symmetries, a light scalar singlet $h$, and the remaining SM particles, but is otherwise completely general.

The main points of our analysis can be summarized as follows.

- We discuss the most general observables arising from the full angular distribution of the 4-lepton final state in $h\rightarrow Z\ell ^+\ell ^-$, $Z\rightarrow \ell ^{'+}\ell ^{'-}$. The nine coefficients $J_i$ describing the angular distribution are expressed through the six form factors $G_{V,A}$, $H_{V,A}$, and $K_{V,A}$.
- Interesting observables, besides the dilepton-mass spectrum $d\Gamma /ds$, can be constructed from the angular distribution. Examples are:
  - The forward–backward asymmetry $A_{\alpha \beta }$ measuring $J_3$ and $B_{\phi }$ measuring $J_6$. These quantities are strongly suppressed in the SM because of the smallness of the vectorial coupling $g_V$. On the other hand, this implies an enhanced relative sensitivity to new physics. The required precision of a few $\%$ might be within reach of the LHC.
  - $J_7$ or $J_9$ give similar information as $d\Gamma /ds$, but should have different experimental systematics because of the characteristic angular dependence associated with them.
  - CP violation in the coupling of $h$ to electroweak bosons is probed by $J_4$, $J_5$, $J_8$, which enter the terms in the decay distribution odd in the angle between the dilepton planes $\phi$. Their effects are, however, expected at the per-mille level and thus out of reach of the LHC.
- The form factors are expressed in terms of the coefficients of the complete effective Lagrangian at next-to-leading order, ${\mathcal {O}}( v^2/\Lambda ^2\sim 1/(16\pi ^2))$. We use the electroweak chiral Lagrangian, extended to include a light Higgs singlet $h$, and take into account all NLO new-physics effects at tree level, including the renormalization of SM fields and parameters. The effective Lagrangian for a linearly realized Higgs is also considered with operators up to dimension 6.
- Based on effective-theory power counting, the potentially dominant impact of new physics arises from the leading-order $hZZ$ coupling $a$, which only affects the overall decay rate, but not the angular and dilepton-mass distributions. The latter can only be modified by the NLO coefficients in the Lagrangian.
- Power counting gives a typical size of the NLO coefficients of $\sim v^2/\Lambda ^2\sim 1\,\%$, up to coupling constants and numerical factors. With this estimate the new-physics effects are typically small. In particular, the contributions of the virtual $Z$ and $\gamma$, which could in principle be inferred from the profiles of the different mass distributions turn out to be at the per-mille level and therefore too small to be detected. Somewhat larger effects (up to 5 %) may be possible in specific scenarios, for instance from enhanced $hZ\bar{l}l$ local couplings $h_{V,A}$ in a strongly interacting Higgs sector. Quantities such as $A_{\alpha \beta }$ and $B_{\phi }$, with their large sensitivity to NP corrections, could be especially interesting in this respect.
- For the quantitative extraction of new-physics coefficients from data, radiative corrections have to be taken into account. To NLO (one loop) in the Standard Model they have been computed in [44, 45].

New-physics effects in $h\rightarrow Z\ell ^+\ell ^-$ decay distributions are expected to be small, even in the case of a strongly interacting Higgs sector. The tree level SM contribution is the dominating effect and NP can potentially show up typically at the percent level. Nevertheless, this NP suppression can be compensated by statistics, and we have shown that interesting opportunities exist for precision measurements, already at the LHC, which could provide valuable insight into electroweak symmetry breaking. The rich subject of $h\rightarrow Z\ell ^+\ell ^-$ observables should therefore be fully explored by experiment.

## Figures and Tables

**Fig. 3:**
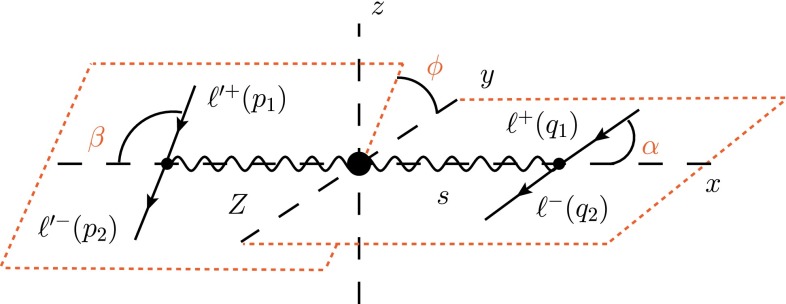
Definition of angles in $h\rightarrow Z\ell ^+\ell ^-$, $Z\rightarrow \ell ^{'+}\ell ^{'-}$

## Appendix A: 4-body decay kinematics

In order to describe the full angular distribution of $h(k)\rightarrow Z(p)\ell ^+(q_1)\ell ^-(q_2)$, followed by $Z(p)\rightarrow \ell ^{'+}(p_1)\ell ^{'-}(p_2)$, one needs to specify four variables. A convenient choice is to select the invariant mass of the dilepton pair, $q^2=(q_1+q_2)^2\equiv M^2_h s$, together with 3 angles $\alpha ,\beta ,\phi$. The angular variables are defined as in [46, 47]: $\alpha$ and $\beta$ are the angles, in the respective dilepton c.m.s., between the ${\ell }^+$ momenta and the direction of the dilepton systems as seen from the Higgs rest frame, while $\phi$ is the angle between the dilepton planes. Referring to the $xyz$-coordinate frame shown in Fig. 3, the precise definition of the angles can be stated as follows:

- The dilepton momentum ${\vec {q}}$ in the rest frame of $h$ defines the direction of the positive $x$-axis, $\vec {e}_x=\vec {q}/|\vec {q}|$.
- $\alpha$ is the angle between $\vec {e}_x$ and the $\ell ^+$ momentum $\vec q_1$ in the $\ell ^+\ell ^-$ c.m.s.
- $\beta$ is the angle between $-\vec {e}_x$ and the $\ell ^{'+}$ momentum $\vec {p}_1$ in the $\ell ^{'+}\ell ^{'-}$ c.m.s.
- $\phi$ is the relative angle between the normals of the decay planes, $\vec {e}_x\times \vec {q}_1/|\vec {e}_x\times \vec {q}_1|$ and $\vec {e}_x\times \vec {p}_1/|\vec {e}_x\times \vec {p}_1|$, counted positive from the former to the latter in the positive direction around $\vec {e}_x$.

In the following we will assume that the final-state leptons are massless, which is a very good approximation at the electroweak scale.

The lepton momenta in the respective dilepton center-of-mass systems can then be parametrized as

33 $$\begin{aligned} q^\mu _{1}&= \frac{M_h\sqrt{s}}{2}\left( 1,{\hat{n}}_1\right) ,\quad q^\mu _{2}=\frac{M_h\sqrt{s}}{2}\left( 1,-{\hat{n}}_1\right) ,\nonumber \\ p^\mu _{1}&= \frac{M_h\sqrt{r}}{2}\left( 1,{\hat{n}}_2\right) ,\quad p^\mu _{2}=\frac{M_h\sqrt{r}}{2}\left( 1,-{\hat{n}}_2\right) , \end{aligned}$$

with the unit vectors

34 $$\begin{aligned} {\hat{n}}_1&= (\cos \alpha ,\sin \alpha ,0),\nonumber \\ {\hat{n}}_2&= (-\cos \beta ,\sin \beta \cos \phi ,\sin \beta \sin \phi ). \end{aligned}$$

The range of the kinematical variables is

35 $$\begin{aligned} 0 \,&\le \, s\, \le \,(1-\sqrt{r})^2 = 0.076,\nonumber \\ 0\,&\le \, \alpha , \beta \, \le \, \pi ,\nonumber \\ 0 \,&\le \, \phi \, \le \, 2\pi . \end{aligned}$$

The momenta can be boosted to the Higgs rest frame with the following velocities:

36 $$\begin{aligned} \beta _q&= \frac{\lambda }{1-r+s},\quad \beta _p=\frac{\lambda }{1+r-s} \end{aligned}$$

where $r$, $s$, and $\lambda$ are defined in (2). The relevant kinematical invariants are then given by

37 $$\begin{aligned}&q_1\cdot p_1=\frac{M^2_h}{8}\Big [\left( 1+c_{\alpha }c_{\beta }\right) g_H+\lambda (c_{\alpha }+c_{\beta })\nonumber \\&\qquad \qquad \quad -2\sqrt{r s}\, s_{\alpha }s_{\beta }c_{\phi }\Big ],\nonumber \\&q_1\cdot p_2=\frac{M^2_h}{8}\Big [\left( 1-c_{\alpha }c_{\beta }\right) g_H+\lambda (c_{\alpha }-c_{\beta })\nonumber \\&\qquad \qquad \quad +2\sqrt{r s}\, s_{\alpha }s_{\beta }c_{\phi }\Big ],\nonumber \\&q_2\cdot p_1=\frac{M^2_h}{8}\Big [\left( 1-c_{\alpha }c_{\beta }\right) g_H-\lambda (c_{\alpha }-c_{\beta })\nonumber \\&\qquad \qquad \quad +2\sqrt{r s}\, s_{\alpha }s_{\beta }c_{\phi }\Big ],\nonumber \\&q_2\cdot p_2=\frac{M^2_h}{8}\Big [\left( 1+c_{\alpha }c_{\beta }\right) g_H-\lambda (c_{\alpha }+c_{\beta })\nonumber \\&\qquad \qquad \quad -2\sqrt{r s}\, s_{\alpha }s_{\beta }c_{\phi }\Big ],\nonumber \\&q_1\cdot q_2=\frac{M^2_h}{2} s,\nonumber \\&p_1\cdot p_2=\frac{M^2_h}{2} r,\nonumber \\&\epsilon _{\mu \nu \lambda \rho }p_1^{\mu }p_2^{\nu }q_1^{\lambda }q_2^{\rho }= \frac{M^4_h}{8}\lambda \sqrt{r s}\, s_{\alpha }s_{\beta }s_{\phi } \end{aligned}$$

where $$g_H\equiv 1-r-s$$, $$c_\chi \equiv \cos \chi $$, $$s_\chi \equiv \sin \chi $$, and $$\epsilon _{0123}=+1$$.
